# Youth-friendly health services for tuberculosis care: an integrative review and implementation science synthesis

**DOI:** 10.1186/s12913-026-14844-0

**Published:** 2026-06-10

**Authors:** Adam Leonard, Elizabeth Di Giacomo, Jason E. Farley, Nicole Salazar-Austin, Leslie A. Enane

**Affiliations:** 1 Center for Infectious Disease and Nursing Innovation (CIDNI), Johns Hopkins School of Nursing, 525 N Wolfe St, Baltimore, MD 21205 USA; 2 Center for Global Nursing, Institute for Global Health Sciences, University of California, 550 16th St, San Francisco, CA 94143 USA; 3 Department of Pediatrics, Johns Hopkins School of Medicine, 733 N Broadway, Baltimore, MD 21205 USA; 4 Department of International Health, Johns Hopkins Bloomberg School of Public Health, 615 N Wolfe St, Baltimore, MD 21205 USA; 5 The Ryan White Center for Pediatric Infectious Disease and Global Health, Department of Pediatrics, Indiana University School of Medicine, 705 Riley Hospital Drive, Indianapolis, IN 46202 USA; 6 Indiana University Center for Global Health, 702 Rotary Circle, Indianapolis, IN 46202 USA

**Keywords:** Adolescent health services, Health service delivery, Implementation science, Tuberculosis, Care engagement

## Abstract

**Background:**

Adolescents and young adults (AYAs) face disproportionate risk of tuberculosis (TB) due to biological vulnerability, mobility, distinct risks for HIV infection and treatment failure, structural barriers, and limited access to adolescent-responsive care. Although the World Health Organization recommends youth-friendly health services (YFHS), implementation in TB care has received little attention. This review synthesizes current evidence on the application of YFHS principles across the TB care continuum to inform models responsive to the needs of AYAs.

**Methods:**

An integrative review was conducted using established methodology and included qualitative, quantitative, and conceptual studies of YFHS in TB care for individuals aged 10 to 24 years in high- and medium-burden countries. Searches were performed in four scientific databases. Two reviewers independently screened records, extracted data, and conducted quality appraisal using Joanna Briggs Institute tools. A deductive thematic synthesis was applied based on the five YFHS domains: for accessible, acceptable, equitable, appropriate, and effective care. Implementation gaps were examined using the integrated Promoting Action on Research Implementation in Health Services (i-PARIHS) framework.

**Results:**

Of 6,067 records screened, 16 studies met inclusion criteria. Most focused on TB treatment, with limited attention to prevention or diagnosis. Strategies to improve accessibility included decentralized services, mobile health tools, and community-based models. Acceptability was related to respectful provider communication, youth-oriented spaces, and privacy protections to reduce stigma. Gaps in equitable care included restrictive consent laws and the absence of tailored services for vulnerable youth. Appropriateness emphasized the importance of integrated care and confidentiality, while effectiveness depended on staff training, clinical readiness, and prompt treatment initiation. Persistent implementation barriers included misaligned policies, fragmented service structures, and limited involvement of youth in service design.

**Conclusions:**

There is an urgent need for adolescent-responsive TB strategies that align service delivery with the preferences and realities of AYAs. Standardized YFHS indicators, meaningful youth engagement, and context-sensitive facilitation approaches are critical to guide implementation. Integrating YFHS principles into national TB programs—supported by dedicated facilitators, cross-sectoral collaboration, and strengthened health systems—has the potential to improve care engagement and outcomes for AYAs living with or at risk of TB.

**Supplementary information:**

The online version contains supplementary material available at 10.1186/s12913-026-14844-0.

Tuberculosis (TB) is the leading infectious cause of death among adolescents and young adults (AYAs) globally, despite this life stage otherwise being associated with optimal health and limited disease burden [1]. AYAs aged 10–24 years are a key TB population due to markedly increasing TB incidence across this age span and particular barriers to service engagement [2, 3]. Yet, evidence is limited on TB epidemiology and treatment outcomes for this age group due to the bifurcation of routine surveillance data into pediatric (ages 14 and under) and adult (ages 15 and older) strata [4]. It has been estimated that there were 1.82 million incident TB cases among AYAs in 2019 [5]. As of 2019, TB incidence was increasing among AYAs in the Africa and Western Pacific World Health Organization (WHO) regions over the previous two decades. Since the COVID-19 pandemic emerged in 2020, further overall increases in global TB incidence across age groups have hampered the TB response [5]. Disproportionate TB incidence among AYAs reflects a convergence of biological, developmental, and structural factors that heighten susceptibility to infection and delay diagnosis. These AYA-specific barriers further contribute to poor treatment outcomes and increased risk for care disengagement among AYAs [6]. Further, high incident HIV infections and risks for virologic failure in this age group increase vulnerability to TB infection and active disease [7].

Health policies, health system design, and service delivery all significantly influence AYAs’ access to and engagement with TB care. The current lack of AYA-specific guidance in national TB service plans and limited integration of TB into national adolescent health plans result in siloed services for AYAs within health systems, and variable quality of TB care [8]. Although the WHO recommends adolescent-responsive TB services, including age-appropriate communication, peer support models, and integration with other youth health services, these recommendations are rarely implemented in practice [9, 10]. Most national programs lack operational guidance or resources to translate these standards into routine service delivery for AYAs [11].

This integrative review aims to analyze and synthesize findings from the published literature on qualitative perspectives, quantitative associations, and expert guidance for implementing youth-friendly health services (YFHS) across the TB care continuum in high- and medium-burden TB countries. The objective is to inform the planning, implementation, and evaluation of innovative TB service delivery models for AYAs to improve outcomes for this population.

## Methods

This integrative review was organized and conducted following the principles set forth by Toronto and Remington [12]. An integrative review serves to provide a comprehensive, rigorous scientific review that broadly evaluates a specific phenomenon by synthesizing empirical research findings (experimental and nonexperimental, qualitative and quantitative) as well as the relevant theoretical and practice literature [13]. To carry out an integrative review on the implementation of YFHS in TB care, a search strategy was developed with a medical informationist to identify empirical, conceptual, and expert opinion sources for review.

### Information sources and search strategy

Searches were conducted in February 2024 in PubMed, Cumulative Index to Nursing and Allied Health Literature (CINAHL) on the EBSCOhost platform, Embase, and Web of Science using natural and control (e.g., MeSH) language combined with Boolean logic, aligned with the Peer Review of Electronic Search Strategies (PRESS) guidelines [14]. Keywords to identify YFHS included ‘adolescent health services’ (MeSH), ‘adolescent friendly’, and ‘youth friendly’. The tuberculosis MeSH term was used, as was the truncated natural language analogue ‘tuberculo*’ for keyword and abstract search (the full search strategy is included in a Supplementary Appendix). The same search was rerun in October 2024 to identify new publications. No publication date range or geographic limitations were applied. Reference lists of exemplar publications were hand-searched to identify additional relevant sources not returned in the previous queries.

Foundational WHO guidance [9, 10, 15, 16] and selected policy analyses [17–20] were reviewed separately to contextualize the scope of the problem, document gaps in adolescent-responsive TB policies, and inform interpretation of findings and recommendations. However, these document-based sources were not included in the analytic synthesis or results tables, as they did not report primary empirical evidence on YFHS implementation in TB care. Their contribution is therefore reflected in the background framing and discussion of the policy–practice gap, rather than in the results of the integrative review.

### Eligibility criteria

Two reviewers independently assessed articles against the a priori inclusion and exclusion criteria. A Population, Intervention, and Outcome (PIO) framework guided eligibility criteria. This defined the Population of AYAs, their caregivers, and healthcare workers (HCWs) providing TB-related services to this population; the Intervention of YFHS, as defined by the WHO across key domains [16]; and care Outcomes across the TB prevention and treatment continuum, including prevention, screening, diagnosis, and treatment. Eligible studies were English-language publications involving AYAs aged 10–24 years receiving TB-related services, their caregivers, or HCWs providing care to this population. Studies including populations outside this age range were included only if disaggregated data for AYAs aged 10–24 years were reported. For example, studies with populations aged, e.g., 0–18 or 15 and above were not included if an AYA age group was not disaggregated in the results. Studies with age ranges within the AYA age range e.g., 10–19 or 15–24 were included. Included studies were limited to those conducted in WHO-defined high- or medium-TB-burden countries [21]. Data were extracted following both the initial search in February 2024 and the repeat search in October 2024. Conference abstracts and case studies were excluded. Reviewer disagreements were resolved through discussion and, when needed, consultation with a senior author.

### Data collection and critical appraisal

The primary author reviewed each selected manuscript and abstracted data using a custom standardized electronic form to collect article details, including publication year, country or countries where the study was conducted, study design, sample composition and size, data collection methods, and main findings. The same author applied the Joanna Briggs Institute’s (JBI) critical appraisal tool relevant to each study design to evaluate the quality and rigor of the work and to inform inclusion decisions [22]. The JBI critical appraisal tools provide a structured approach to evaluate the trustworthiness, relevance, and integrity of study findings for each study design [22]. In addition to study-level critical appraisal, potential publication bias was assessed at the body-of-evidence level using systematic, indirect assessment methods appropriate for integrative reviews [12]. Because statistical tests were not applicable, bias was evaluated by examining search breadth; the distribution of evidence across TB care stages, populations, and YFHS domains; the representation of null or unfavorable findings; and reliance on specific study designs, with evidence gaps documented.

### Analysis and synthesis methods

A three-step approach was used to synthesize findings from both qualitative and quantitative studies, guided by the five WHO YFHS domains (Table 1) [16]. First, all extracted data were organized into a structured matrix, with each row representing a study and columns capturing study characteristics, populations, methods, outcomes, and content relevant to the YFHS domains [23].

**Table 1:** World health organization youth-friendly health services domains

WHO YFHS Domain	WHO Definition	Included Characteristic(s)	Highlighted Practices for Operationalizing YFHS in TB Care
Accessible	Adolescents are able to obtain the health services that are available	• Cost • Clinic location • Hours	• Mobile clinics • Afterschool hours • Free services
Acceptable	Adolescents are willing to obtain the health services that are available.	• Staff attitude • Waiting time • Physical environment	• Youth-only waiting area • Drop-in services • Welcoming staff
Equitable	All adolescents, not just selected groups, are able to obtain the health services that are available.	• Inclusive policies and practices	• HCW cultural sensitivity trainings • Inclusive signage
Appropriate	The right health services (i.e. the ones they need) are provided to them.	• Confidentiality • Service integration	• Private consultation rooms • Whole-person care
Effective	The right health services are provided in the right way, and make a positive contribution to their health.	• Clinical competencies • Positive health outcomes	• Adolescent health trained HCWs • System evaluation metrics tied to leading AYA morbidity and mortality data

Based on World Health Organization, Making health services adolescent friendly: developing national quality standards for adolescent friendly health services, Geneva: 2012

*AYA: Adolescents and Young Adults; HCWs: Healthcare Workers; TB: Tuberculosis; WHO: World Health Organization; YFHS: Youth-Friendly Health Services

Second, a deductive coding process was applied, using the five YFHS domains as a priori analytic categories to thematically code findings across studies. Because WHO YFHS domains are conceptually related rather than mutually exclusive, some service characteristics (e.g., same-day care or youth-designated spaces) were relevant to more than one domain depending on whether they influenced accessibility, acceptability, or effectiveness. To enhance analytic specificity, we consulted Ambresin et al.’s synthesis of youth perspectives on healthcare quality to inform subthemes within each YFHS domain, drawing on their defined domains and indicators of youth-friendly care to inform our own [24]. Quantitative data were interpreted in light of their relevance to domain constructs, while qualitative findings were coded into thematic subcategories aligned to each domain. Third, cross-study synthesis was conducted within each domain to identify recurring patterns, areas of convergence and divergence, and notable evidence gaps. This process facilitated an integrated narrative that reflects the full scope of evidence across methodological approaches.

To guide interpretation of the synthesized findings and support actionable recommendations from this review, we applied the integrated Promoting Action on Research Implementation in Health Services (i-PARIHS) framework to structure the discussion. i-PARIHS is a widely used implementation science framework that conceptualizes successful implementation as a function of the dynamic interplay between the nature of the intervention (*innovation*), the individuals and teams involved (*recipients*), the setting or system (*context*), and the presence of skilled facilitation (*facilitation*) to support uptake [25]. This framework was selected due to its strength in bridging evidence and practice by emphasizing contextual readiness, recipient engagement, and tailored facilitation strategies. Given the multisectoral and policy-sensitive nature of youth-friendly TB services, i-PARIHS offers a structured lens to identify leverage points for strengthening implementation. This approach can also inform future implementation research, policy development, and adaptation of interventions aimed at improving TB outcomes among AYAs.

## Results

**Fig. 1:**
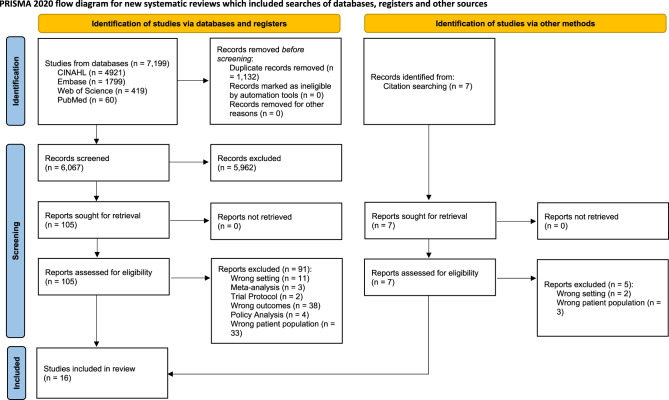
PRISMA flow diagram. Description: PRISMA flow diagram illustrating the number of studies by identification, screening, and inclusion stages

The search criteria returned 6067 deduplicated results across the four databases for screening (Fig. 1). After initial screening, 105 studies remained and were advanced to full-text review. An additional seven studies identified through citation searching were reviewed. In total, 16 studies met the full inclusion criteria and are included in this review.

### Characteristics of included studies

The 16 included studies varied in design, focus along the TB care continuum, and study populations (Tables 2 and 3). Key characteristics are summarized as follows:

- Study design: Most studies were qualitative (*n* = 10), with fewer quantitative studies employing observational (*n* = 3) or quasi-experimental (*n* = 3) designs.
- TB care phase: More than half of studies focused on TB treatment (*n* = 10), while comparatively few examined TB screening (*n* = 4), diagnosis (*n* = 3), or tuberculosis preventive treatment (TPT) (*n* = 4).
- HIV status: Among the 13 studies that included adolescent and young adult (AYA) participants, nearly half included mixed HIV-status samples (*n* = 6), while only two studies—both focused on TPT—exclusively enrolled youth living with HIV.
- Drug resistance: Of the treatment-focused studies, only one addressed drug-resistant TB; the remainder focused on drug-susceptible TB or did not specify resistance status.

These study characteristics provide context for the thematic synthesis below, which integrates findings across designs and settings using the WHO-defined YFHS domains.

**Table 2:** Qualitative article summaries

Author Year and TB Care Domain	Country	YFHS Domains	Design	Sample (n) YLWH (%)	Results
Amuge 2024 *TPT*	Uganda	Accessible Acceptable Appropriate Effective	Qualitative	AYAs (20), HCWs (10), PCGs (20) *YLWH (100%)*	Facilitators: **1. Individual Level**: ◦ Perceived risk of TB and desire to avoid the disease. ◦ Previous experience with TB treatment motivated adherence. ◦ Desire for health preservation. **2. Interpersonal Level**: ◦ Support and reminders from family members and caretakers. ◦ Supervision and encouragement from parents. **3. Community Level**: ◦ Guidance, counseling, and mass awareness campaigns about TPT. ◦ Information provided by health workers about TPT benefits and side effects. **4. Institutional Level**: ◦ Integration of TPT and ART refill visits to reduce clinic visits. ◦ Community-based drug delivery models. ◦ Follow-up mechanisms like phone calls and reminders. **5. Structural Level**: ◦ National policies and performance indicators promoting TPT uptake. ◦ Regular audits to identify gaps in TPT prescription and delivery. Barriers: **1. Individual Level**: ◦ TB and HIV-related stigma. ◦ Fear of drug side effects and pill burden. ◦ Fatigue from taking medication when not sick. ◦ Financial constraints and lack of food to take with medication. **2. Interpersonal Level**: ◦ Lack of parental support and supervision. ◦ Non-disclosure of HIV status to children. ◦ Restrictions by spouses preventing clinic visits. **3. Community Level**: ◦ Misconceptions about TPT benefits and side effects. ◦ Stigma in schools and communities. **4. Institutional Level**: ◦ Long waiting times at clinics. ◦ Rude or unapproachable health workers. ◦ Missed opportunities due to health workers forgetting to prescribe TPT. ◦ Drug stock-outs. **5. Structural Level**: ◦ Long distances to clinics and high transport costs.
Chiang 2023 *DSTB Treatment*	Peru	Accessible Acceptable Equitable Appropriate	Qualitative	AYAs (34), HCWs (15), PCGs (34) *YLWH (6%)*	AYAs/PCGs reported barriers: • Daily in-person DOT administered during limited hours. • Inflexible policies for take-home DOT or alternate clinic DOT when traveling or for AYAs with divorced/separated parents. • Infrequent, but reported disrespectful treatment by HCWs. • Very few AYAs complained about long wait times at PHCs. • Public waiting areas for TB treatment violated privacy and confidentiality resulting in anticipated stigma among AYAs. HCWs reported barriers: • DOT hours conflicted with AYAs/PCGs school or work schedules.
Chiang 2023 *Screening* *TPT* *Treatment*	High Burden Countries generally	Accessible Acceptable Equitable Appropriate Effective	Expert Opinion	AYAs (10), HCWs (24)	Recommendations for HCWs: • Regular training and competency evaluation for HCWs caring for AYAs, focusing on confidentiality, nonjudgmental care, and destigmatizing language. • Increase awareness of AYAs’ TB risk and appropriate screening, diagnostic, and referral practices. • Tailor health education to be accessible to AYAs and their PCGs. • Address psychosocial and mental health needs of AYAs with TB, including screening and care for mental health and SUDs. Recommendations for PHCs: • Offer after-school and weekend clinic hours. • Minimize AYA clinic wait times. • Decentralize TB care for AYAs into community settings. • Improve care transfer processes for AYAs who need to relocate. • Integrate TB care as part of comprehensive AYA care (e.g., SRH, mental health, SUD). • Utilization of peer counselors and peer support groups.
Das 2021 *DRTB Treatment*	India	Acceptable Appropriate Effective	Qualitative	AYAs (6), HCWs (8), PCGs (5) *YLWH (0%)*	AYA/PCG reported barriers: • Minimal communication with HCWs about their TB illness; hyperfocus on adverse events only. • Apathetic attitude of some HCWs. • Irregular feedback from HCWs during treatment. • Insufficient information regarding DR-TB disease and treatment. • Tedious paper-based monthly adherence monitoring forms. HCW reported barriers: • Inexistent age-adapted counseling tools. • Lack of AYA friendly clinic spaces.
Enane 2020 *Treatment*	Botswana	Accessible Acceptable Appropriate	Qualitative	HCWs (16)	HCW reported needs for YFHS: • Flexible clinic hours around AYAs’ school and work schedules. • Non-judgmental care. • More intensive counseling from HCWs to build trust. HCW reported barriers to YFHS: • Lack of confidentiality and concern about stigma. • Understaffed or overburdened clinics. • Long waiting times. • Lack of resources to offer financial or nutritional support.
Laycock 2021 *Treatment*	Botswana	Accessible Acceptable Equitable Appropriate Effective	Qualitative	HCWs (16)	Equitable • Younger AYAs require parental consent for care. • Non-citizens unable to access treatment at the time of interviews. Accessible • Free or affordable care services. • Convenient hours • Information about SRH services and how to access them. • Community support for AYA to obtain services. • Pervasive stigma is a central barrier to care for AYA. • Need for social and family support for AYA in TB care. • Community-based DOT supports care access for AYA. • Interest in peer support models for TB care. Acceptable • Confidentiality and privacy with services. • Non-judgmental, friendly HCWs. • Can access care with or without an appointment. • Appealing care environments and dedicated spaces for AYAs. Appropriate • Need integrated services for MH, SUD. • Need material supports for AYAs with food and/or financial insecurity. Effective • HCW competencies to work with AYAs. • Evidence-based protocols and guidelines for AYA TB. • Increased visit time to work effectively with AYAs. • Requires adequate staffing and material resources to meet AYA needs, e.g. through intensive counseling and home visits.
Muttamba 2021 *Treatment*	Uganda	Acceptable Appropriate Effective	Qualitative	AYAs (32), HCWs (34) *YLWH (not stated)*	AYA reported barriers: • Encountering HCWs who were unfriendly, judgmental, or uncooperative. • HCWs do not explain procedures to AYAs. • HCWs prescribe treatment that AYAs must buy. • AYAs felt health workers judged them unfairly for delaying visits to the health facility. • AYAs described the attitude of health workers as demotivating. • AYAs noted they must forego school or come to health facilities during holidays. • Disappointment with not receiving full care needed and are occasionally asked to return to the health facilities later. • Confidentiality concerns with having to carry around sputum mugs to provide a sample. HCW reported barriers: • Many HCWs may miss TB in AYAs because they associate TB with HIV and lowered immunity, which they did not believe to be common in AYAs.
Strauss 2023 *TPT*	South Africa	Accessible Acceptable Appropriate	Mixed Methods	AYAs (170 quant, 5 qual), HCWs (5 qual), PCGs (173 quant, 5 qual) *YLWH (7%)*	**AYA preferences:** • Preferred shorter regimens with fewer clinic visits, local clinic-based care, and shorter waiting times. • Avoided community-based care due to stigma concerns. • Side effects were a major concern, especially if noticeable to others. • Shorter regimens were preferred but only if combined with fewer clinic visits. • Adolescents prioritized stigma avoidance and health system attributes over pill size and taste. Parent/Caregiver preferences: • Valued monthly clinic visits for adherence support and additional care. HCW insights: • **Location of Services**: • Suggested community healthcare worker home visits to make accessing TPT easier, as some patients avoid clinics. • **Waiting Time**: Acknowledged that 15-minute waiting times would be ideal but unrealistic due to overcrowded clinics. They considered 1.5 to 3 hours as more practical.
Suwary 2024 *Treatment*	Papua New Guinea	Acceptable Appropriate	Qualitative	AYAs (54) *YLWH (2%)*	AYA reported barriers: • Some AYAs felt the attitude of the healthcare workers to them was on occasion demeaning (*n* = 5) or unfriendly (*n* = 2). • Some AYAs commented that HCWs talked only to parents. • No perceived communication about diagnosis or management plans, only direction (*n* = 8). • AYAs reported feeling uncomfortable around other older or younger patients (*n* = 17). • Many used the phrase feeling “out of place” amongst older men or women or small infants. • Some AYAs found this experience of being out of place to be distressing. AYA reported facilitators: • AYAs reported HCWs talked to them in ways that they understood and felt were appropriate (*n* = 42). • Most expressed they trusted the health information from HCWs. • Most AYAs endorsed a preference for a peer-focused or adolescent-friendly setting, stating that it would allow for positive peer-interaction, and improve their experience and the quality of health care received (*n* = 44).
Zvonareva 2021 *DSTB Treatment*	Russia	Acceptable Appropriate	Qualitative	AYAs (8), HCWs (3), PCGs (6) *YLWH (not stated)*	AYA/PCG reported facilitators: • Prompt and comprehensive information sharing from HCWs. • Clear treatment education from HCW on hospital admission. • Respect for AYA autonomy during treatment. • Open communication channels with HCWs. HCW reported facilitators: • Patient-centered care planning and decision-making. • Clear discussion of treatment plan and prognosis with frequent updates. • Conveying openness to AYA-initiated communication on care.

*Acronyms*: AYAs – Adolescents and Young Adults; HCWs –Health Care Workers; DOT – Direct Observation of Therapy; DRTB – Drug ResistantTB; DSTB – Drug Susceptible TB; PCGs – Parents/Caregivers; PHCs – primary healthcare centers; TPT – Tuberculosis Preventive Treatment; YFHS – Youth-Friendly Health Services; YLWH – Youth Living with HIV

YLWH percentage calculated from reported data in each study; ‘not stated’ no data on youth HIV status was provided in the manuscript

**Table 3:** Quantitative article summaries

Author and Year	Country	YFHS Domains	Design	Sample (n) YLWH (%)	Results
Black 2023 *TPT*	Kenya	Accessible Acceptable Effective	Cohort	AYAs (10,328), HCWs (86) *YLWH (100%)*	• TPT Initiation: higher at clinics with lower patient-to-staff ratios (*p* = 0.044) and with designated AYA areas (*p* = 0.001). • TPT Completion: higher at dispensaries compared with all other clinic types (*p* = 0.035) and at clinics with lower patient-to-staff ratios (*p* = 0.004).
Omongot 2023 *Screening*	Uganda	Acceptable	Quasi-Experimental	AYAs (394) *YLWH (15%, 33% unknown HIV status)*	TB Care Continuum: • There was an increase in the numbers of AYAs screened for TB after the intervention across all the four project health facilities (average increase of 94%, 1.9 IRR, 95% CI 1.9–2.0, *p* < 0.001). • There was an increase in the average number of presumptive TB AYAs identified during the project period (average increase of 123%, 2.2 IRR, 95% CI 1.9–2.5, *p* < 0.001). • There was an increase in the average number of AYAs tested for TB after the intervention (average increase of 250%, 3.3 IRR, 95% CI 2.8–3.8, *p* < 0.001). • There was no significant average increase in the number of TB positive AYAs identified after the intervention (average increase of 50%, *p* = 0.170). • There was no significant improvement in linkage of adolescents to treatment and care (average increase of 50%, *p* = 0.154).
Reif 2016 *Screening*	Haiti	Accessible Acceptable Appropriate	Cohort	AYAs (3,483) *YLWH (3%)*	TB Care Continuum: • Among those who presented for HIV testing, 168 reported symptoms of TB and among symptomatic participants, 5% (13/168) tested sputum positive for TB disease (rate of 338/100,000 persons among all screened). • No AYAs reported fears of loss of confidentiality with using outreach services.
Reif 2018 *Treatment*	Haiti	Accessible Acceptable Effective	Cohort	AYAs (1,005) *YLWH (7%)*	• 93% (791/846) of AYAs who started anti-TB treatment initiated medication on the same day they presented with symptoms based on clinical suspicion and before the results of confirmatory testing. • The median time to initiation was 12 days (interquartile range 7–19) among AYAs who did not start treatment the same day as presentation. • A higher proportion of patients who received same day treatment (624/791, 79%) than those who initiated treatment after the day of presentation (38/55, 69%) had successful TB treatment outcomes (*p* = 0.09).
Sodhi 2024 *Treatment*	India	Accessible Effective	Quasi-Experimental	AYAs (989) *YLWH (not stated)*	• Patients ages 16–24 enrolled in the Connect for Life (CfL) mHealth pilot received more follow-ups from treatment coordinators compared to those not enrolled. This increased engagement was linked to better treatment adherence. • Patients aged 16–19 and 20–45 who used the CfL application had a higher likelihood of successful treatment outcomes (completion or cure) compared to their counterparts who did not use the application.
Szkwarko 2016 *Screening*	Kenya	Accessible Acceptable	Quasi-Experimental	AYAs (116) *YLWH (not stated)*	• TB Care Continuum: Of 114 (98%) SCY with a positive screening questionnaire, 104 (90%) provided a first spot sputum sample, while only 39 (34%) provided a morning sputum sample. The one street youth who tested smear-positive for TB tested negative for HIV upon linkage to care and completed anti-TB treatment successfully.

*Acronyms*: AYAs – Adolescents and Young Adults; HCWs –Health Care Workers; IRR – Incident Rate Ratio; PCGs – Parents/Caregivers; SCY – Street Connected Youth; TPT – Tuberculosis Preventive Therapy; YFHS –Youth-Friendly Health Services; YLWH – Youth Living with HIV

YLWH percentage calculated from reported data in each study; ‘not stated’ no data on youth HIV status was provided in the manuscript

### YFHS themes

Study findings and recommendations across all 16 reviewed publications are synthesized below. All five domains of the WHO-defined YFHS were addressed in the studies. The acceptability domain of YFHS was the most frequently addressed concept across studies, while the equitable domain was the least specifically addressed. Across domains, reported facilitators and barriers reflected challenges related to recipient engagement (e.g., adolescent and HCW attitudes and capabilities), contextual constraints (e.g., staffing, service organization, and policy environments), and the availability of implementation supports, linking to the multi-level dynamics explored in the discussion using the i-PARIHS framework.

#### Accessible

Findings consistently emphasized ways in which clinic cost, distance, and hours affected AYAs’ access to TB care. Structural barriers like long travel distances, high transport costs, and inflexible clinic hours interfered with school and work [26–28]. Community-based services, decentralized models, and reduced clinic visits improved uptake in intervention studies and were reported as key facilitators across qualitative findings [29–32]. Similarly, same-day TB treatment initiation based on symptoms and clinical suspicion increases uptake and treatment completion compared to treatment initiated after test results are received (often up to two weeks later) [33]. Consistent with this, cohort studies reported higher treatment success with same-day TB treatment initiation and higher TPT initiation and completion at clinics with lower patient-to-staff ratios [30]. Mobile health (mHealth) tools facilitated greater access to clinic appointments and follow-up care [34]. Requirements for clinic-based directly observed therapy (DOT) was a notable factor limiting TB service accessibility for AYAs as were inflexible care transfer practices. Active TB case-finding in non-clinical community settings where AYAs already engage increased access to care [35, 36]. These access-related barriers and facilitators primarily reflected contextual conditions, including service organization, resource availability, and system-level flexibility, which shaped the feasibility of implementing youth-friendly adaptations.

#### Acceptable

Acceptability depended on commonly-described factors such as HCW attitudes, wait times, and youth-friendly environments. AYAs cited disrespectful or apathetic HCW interactions as demotivating, while friendly, respectful providers improved trust [26, 29, 37–39]. Participants recommended dedicated AYA spaces, shorter wait times, and flexible same-day service options to improve service acceptability [27, 32, 33, 35, 36, 39]. Clinics with designated AYA waiting areas had higher TPT initiation and completion rates [30]. Some AYAs reported feeling “out of place” in mixed-age waiting areas, and non-private DOT procedures in the clinic were linked to anticipated stigma [28, 39]. Both AYAs and HCWs favored peer-supported and outpatient TB treatment models, while hospitalization for TB treatment was considered unacceptable unless medically necessary [38, 39]. Training HCWs in youth-friendly care and shared decision-making was a widely supported recommendation along with youth-focused messaging for TB education [31, 40, 41]. Notably, these findings centered on recipient-level factors, particularly adolescent perceptions and health care worker attitudes, highlighting how interpersonal dynamics influenced engagement with TB services.

#### Equitable

Equitable care was infrequently examined as an explicit youth-friendly health services (YFHS) domain; however, multiple studies reported implicit equity-related findings that reveal systematic barriers to accessing TB services. Several publications highlighted challenges faced by acutely vulnerable subgroups, including migrant youth, non-citizens, child laborers, and street-connected adolescents, for whom legal status, mobility, and lack of formal caregivers constrained access to routine TB care [31, 32, 35, 42]. Requirements for parental or guardian consent emerged as a recurrent barrier for younger adolescents, including youth who traveled between homes of separated parents, effectively excluding those without available or supportive caregivers from timely diagnosis, prevention, or treatment [28, 32].

Across studies, sampling approaches often underrepresented younger adolescents and vulnerable AYAs (e.g., disconnected from school), limiting insight into age- and situation-specific barriers and suggesting potential inequities in both service access and evidence generation. Collectively, these findings indicate that equity-related constraints were embedded in programmatic and research practices, yet were infrequently labeled or analyzed as equity concerns in the included studies. These equity-related constraints reflected interactions between recipient vulnerability and contextual factors, including legal frameworks, service eligibility criteria, and research practices, which shaped who could access care and whose experiences were represented [27, 36, 38].

#### Appropriate

Appropriateness was characterized in relation to service integration and confidentiality. Recommended approaches to AYA TB care called for holistic care that integrated services for TB with those for sexual and reproductive healthcare (SRH), HIV, mental health, and substance use disorder services [31, 32]. Similarly, economic and nutrition support services were also seen as necessary and appropriate components of AYA TB care [26, 31, 32]. Care aspects that could lead to disclosure of TB illness, such as sputum cup transport, DOT, and disease-specific clinics, were viewed as undermining confidentiality and stigmatizing [26–29, 38]. Integration of TB screening into general preventive care services helped preserve privacy and normalize TB testing [36]. AYA-centered communication and the inclusion of AYAs’ perspectives in healthcare decision-making were viewed positively as foundational to the YFHS model [39, 41]. The lack of AYA-specific clinical tools for TB prevention and treatment counseling and education was also noted as a shortcoming [37]. Together, these findings point to gaps in the design and adaptation of care models, underscoring innovation-level challenges in aligning TB services with the holistic needs of adolescents and young adults.

#### Effective

Effectiveness was characterized by timeliness, clinical competency, and structural readiness for TB services. Same-day treatment initiation and community-based delivery were associated with better TB screening uptake and improved TPT completion in two cohort studies [30, 33]. Several youth-responsive service characteristics previously described under accessibility and acceptability domains—including lower patient-to-staff ratios, designated youth areas, and mHealth-supported follow-up—were associated with improved treatment completion outcomes [30, 34]. However, limited staffing, poor coordination, and lack of youth-focused tools undermined effectiveness [26, 32]. For example, one study reported that HCWs’ assumptions linking TB primarily to HIV contributed to missed or delayed TB diagnoses among adolescents, illustrating how gaps in clinical competency can undermine service effectiveness [38].

Recommendations included establishing AYA-specific protocols, expanding HCW training, and ensuring adequate time and resources per visit [31, 32, 38]. Effective services were also those that incorporated quality assurance feedback mechanisms and had performance indicators to track gaps in TB care delivery [29]. Variation in effectiveness was closely tied to contextual readiness and the presence or absence of structured implementation supports, suggesting a critical role for facilitation in translating youth-friendly principles into routine practice.

### Study quality and risk of bias

No studies were excluded based on critical appraisal. Application of the JBI appraisal tools identified recurring methodological limitations across qualitative, observational, and quasi-experimental studies (see Supplementary Appendix for JBI Assessment results). Selection bias was most common among qualitative and cohort studies, as participants were frequently recruited through existing clinic engagement, TB treatment programs, or HIV care services, potentially underrepresenting AYAs who were disengaged from care or unable to access health facilities. This was particularly evident in studies recruiting exclusively from clinic-attending populations or ongoing treatment cohorts, including studies focused on TPT delivery among youth living with HIV and facility-based TB treatment experiences. Although a small number of studies attempted outreach-based or community-based recruitment, such approaches were less common.

Measurement bias was also common across studies due to inconsistent operationalization of YFHS domains and reliance on non-standardized measures of youth-friendliness, acceptability, or care engagement. Most qualitative studies examined isolated aspects of YFHS, such as provider attitudes, confidentiality, or clinic environment, rather than applying validated or comprehensive YFHS assessment frameworks. Quantitative studies similarly relied on heterogeneous proxy indicators, including patient-to-staff ratios, designated adolescent clinic areas, same-day treatment initiation, or mHealth follow-up engagement, limiting comparability across settings and interventions. Few studies used standardized adolescent health service measurement tools or explicitly grounded their evaluations in established YFHS frameworks.

Potential publication bias also cannot be excluded, as the evidence base was dominated by descriptive qualitative studies and small-scale implementation or pilot evaluations, with limited reporting of null or unsuccessful interventions. In addition, studies from non-English-speaking settings and unpublished programmatic evaluations may have been underrepresented. Collectively, these limitations indicate that the current evidence base remains more exploratory and descriptive than definitive, underscoring the need for methodologically rigorous, equity-oriented implementation research evaluating youth-responsive TB care models across the full TB care continuum.

## Discussion

This review synthesized evidence from 16 studies to examine the current evidence around application of YFHS in TB care for AYAs. Findings revealed consistent structural and interpersonal barriers experienced by youth across the TB care continuum, with the greatest emphasis on needs for greater acceptability and accessibility, and comparatively limited attention to equitable care. Wide research gaps were noted, and as a result, sparse evidence is available to inform YFHS for TB care delivery. Very few studies examined interventions or models for integrating adolescent-specific needs in TB service delivery. Further, there was limited research in youth TPT and TB diagnosis stages of care. Recommendations from the available literature spanned structural, provider, and service delivery levels. Overall, the existing evidence base is largely hypothesis-generating, driven primarily by qualitative and descriptive studies, with limited hypothesis-testing evidence from rigorously designed intervention or efficacy trials.

Gender inequities were infrequently examined and few studies explicitly considered how gender intersects with other axes of disadvantage, such as age, HIV status, migration, poverty, or caregiving roles, to shape access to and engagement with TB services. This limited attention to intersectional inequities highlights gaps in alignment with the WHO AYA-responsive health system framework, which emphasizes the compounding effects of social and structural vulnerability and the need for equitable service provision.

Across countries, common themes emerged, including the importance of confidentiality, respectful provider interactions, and flexible service delivery in supporting adolescent TB care. Stigma and structural barriers, such as long wait times or transport costs, were also consistently reported. However, due to the limited availability of studies in each setting, there was a restricted capacity to assess or compare regional differences fully.

Overall, though, evidence regarding effective interventions and implementation strategies remains low. Despite WHO guidance calling for youth-responsive approaches to TB care, few studies have rigorously evaluated what works, for whom, and under what conditions across the TB care continuum. The literature remains heavily skewed toward descriptive accounts, with a notable lack of well-designed trials or implementation studies tailored explicitly to AYA populations. This evidence gap limits the ability of national TB programs to scale up youth-friendly models of care with confidence. To advance progress, future work must prioritize both rigorous implementation research and concurrent intervention delivery to ensure that promising approaches are tested, refined, and integrated into policy and practice.

**Fig. 2:**
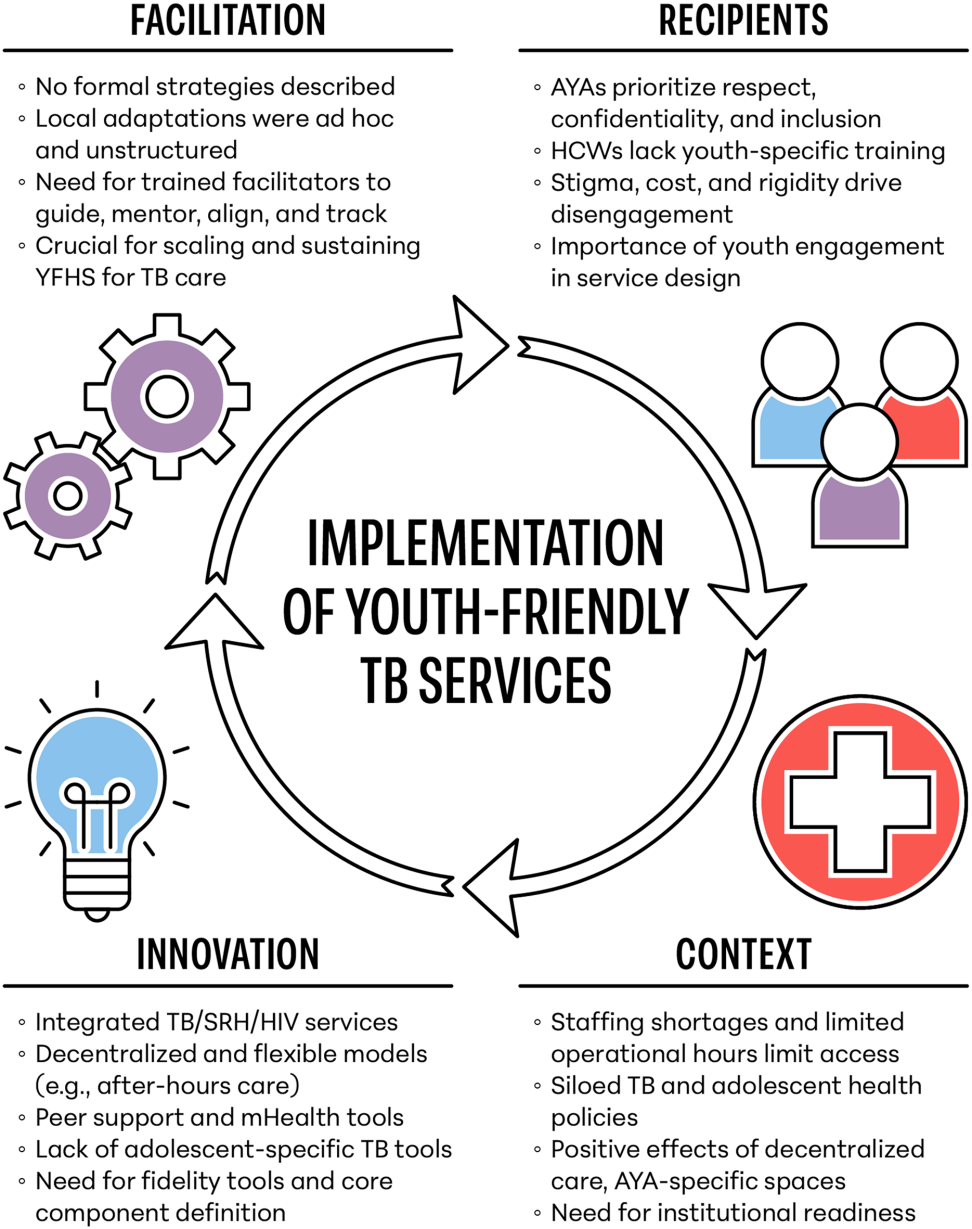
Recommendations for YFHS application to the TB care continuum organized by the i-PARIHS framework. Description: illustrated figure summarizing findings and recommendations for YFHS implementation in TB across the i-PARIHS domains: facilitation, recipients, innovation, and context. Framework from: Harvey G, Kitson A. PARIHS revisited: from heuristic to integrated framework for the successful implementation of knowledge into practice. Implement Sci. 2015;11(1):33. Abbreviations: AYA: adolescents and young adults; HCW: healthcare workers; HIV: human immunodeficiency virus; i-PARIHS: integrated Promoting Action on research implementation in health Services; SRH: sexual and reproductive health; TB: tuberculosis; YFHS: youth-friendly health services

To interpret the synthesized evidence in a structured and theory-informed manner, we applied the i-PARIHS framework to guide our discussion (Fig. 2). This framework identifies four core constructs—innovation, recipients, context, and facilitation—as key determinants of implementation success. The following sections examine each construct in detail, analyzing the current state of evidence and identifying areas for action to strengthen the implementation of YFHS in TB care settings.

### Innovation

The need for innovation in youth-friendly TB care was evident across several studies, with approaches such as integrated TB, SRH, and HIV services; decentralized or community-based models; flexible clinic hours; and peer support, all of which were linked to greater perceived health system acceptability and accessibility, as well as demonstrated adherence. Despite clear needs and preferences for youth-friendly innovations, wide gaps remain in the availability of adolescent-specific interventions, care tools, protocols, and guidance in high-burden settings.

This dearth of evidence from real-world implementation of youth-friendly TB care contrasts with the relatively robust evidence base and implementation experience for YFHS applied to HIV. Research has demonstrated the positive impact YFHS has on HIV testing [43], ART adherence [44], retention in care [45], and transition to adult care [46]. Moreover, studies have also evaluated essential implementation outcomes, identifying both key barriers [47] to and facilitators [48] of YFHS integration into HIV care. The evidence base for interventions to improve YFHS for TB care delivery is, by comparison, sparse. To the extent that funding barriers or other structural barriers to research or care innovations for youth with TB contribute to this disparity, these must be explored and overcome to move towards delivery of evidence-based care for youth with TB.

To advance implementation, there is an urgent need to co-develop adolescent-responsive and scalable innovations with youth input, including tailored communication strategies, service adaptations like mHealth interventions or stigma-reducing delivery models, and youth-focused delivery of novel regimens such as shorter-course TPT. Future work should also define the core components of youth-friendly TB care and establish validated tools to assess fidelity and effectiveness of these innovations across diverse health system contexts.

### Recipients

AYAs consistently emphasized the importance of respectful communication, confidentiality, and inclusive clinic environments in shaping their engagement with TB care. However, HCWs often lacked training in adolescent-responsive approaches, leading to mismatches between service delivery and youth preferences. Structural and interpersonal barriers, including stigma, transportation costs, economic or academic hardships due to missed work or school, and inflexible care models, were commonly cited as drivers of care disengagement. To improve implementation success, recipient-level strategies must address these intersecting needs. This includes equipping HCWs with training in youth-centered communication and care practices, creating supportive peer and family systems, and developing interventions to reduce stigma. Notably, gender-based disparities in TB care—well documented among adults—require closer examination within the AYA context, where social norms, developmental transitions, and differential experiences of autonomy may shape access, engagement, and outcomes in gendered ways. Future research should explicitly evaluate gender inequities in TB services among AYAs to inform more tailored and equitable implementation approaches. Crucially, meaningful engagement of youth in the design and delivery of TB services is essential to ensure services are responsive, accessible, acceptable, and effective.

Incorporating youth participation in TB service design is essential to ensure that interventions consider the lived experiences, preferences, and structural realities of AYAs. Evidence from HIV and SRH programs demonstrates that participatory approaches, such as youth advisory boards, peer-led service models, and human-centered design, can improve service relevance, increase trust, and enhance uptake among youth populations [49, 50]. These approaches shift implementation dynamics by positioning AYAs not as passive recipients but as active partners in care transformation. In the context of TB, similar strategies could help bridge gaps between AYA needs and existing service structures, particularly in settings where misalignment between youth preferences and HCW norms undermines engagement. Meaningful youth involvement also aligns with WHO guidance on adolescent-responsive health systems, which calls for youth engagement in policy, program, and service-level decision-making [15].

### Context

Contextual barriers significantly shape the feasibility and sustainability of implementing YFHS for TB care. Structural limitations such as inadequate clinic staffing, rigid DOT requirements, and restrictive consent laws constrain adolescent engagement and compromise care continuity. Furthermore, the lack of adolescent-specific guidance in national TB strategies reflects a broader misalignment between youth health and TB program priorities. The limited quantitative evidence from the review indicated that decentralized models of care, smaller HCW-to-patient ratios, and clinics with youth-specific waiting areas were associated with improved uptake and treatment adherence.

To support implementation, national TB programs must prioritize the integration of adolescent health principles into TB strategies, revise legal and policy constraints that restrict youth autonomy, and invest in service models that promote accessibility and continuity. Strengthening institutional readiness, including adequate human resources, physical space, and operational policies, is critical to advancing and sustaining YFHS for TB.

Successful implementation of YFHS for TB care requires not only clinic-level adaptations but also broader health system readiness. Governance structures must be responsive to adolescent health priorities, ensuring that national TB programs include accountability mechanisms for adolescent-specific outcomes and integrate youth-responsive indicators into monitoring systems [19]. Intersectoral collaboration—especially between TB, HIV, and adolescent health sectors—is essential to avoid service fragmentation and to support integrated, holistic models of care [20]. However, siloed funding streams and uncoordinated programming remain common barriers.

National TB strategies should preferably include clear policy commitments to decentralization and youth-specific adaptations, supported by legal and regulatory frameworks that affirm adolescent access and reduce structural barriers to access [20]. Investments in institutional readiness, including staffing, infrastructure, and implementation support, are necessary to translate policy into practice. Routine health information systems should be strengthened to enable the collection of disaggregated data by age and sex, facilitating the identification of service gaps and the tailoring of interventions to AYAs’ needs [51]. All of the above considerations and adaptations necessitate dedicated investment and funding. Economic evaluation studies should be undertaken alongside the implementation of scalable, high-quality YFHS to demonstrate whether such investments may indeed be cost-effective for improving TB treatment outcomes and reducing morbidity and mortality associated with TB. Such evidence could bolster political support for greater YFHS in national TB plans.

### Facilitation

Facilitation emerged as a significant gap across the reviewed studies, with no publications describing formal implementation strategies or designated facilitators to support the integration of YFHS into TB care. Adaptations to improve youth responsiveness were often informal, fragmented, and driven by local initiative rather than structured implementation support. This absence of facilitation capacity hampers scale-up and limits the sustainability of promising YFHS models. Future implementation efforts should prioritize the inclusion of dedicated facilitators to guide local adaptation, mentor staff, and coordinate policy alignment and quality improvement processes. Facilitators play a crucial role in helping clinical sites embed YFHS approaches into existing TB service platforms, ensuring consistent application of youth-centered principles, and tracking progress toward improved engagement and health outcomes for adolescents and young adults.

The role of the facilitator is central in implementation science, particularly in operationalizing complex, multi-level interventions like YFHS within disease-specific care environments. Facilitators support implementation by enabling behavior change, building capacity, and aligning innovation, context, and recipients [25]. In the field of youth HIV care, trained facilitators have been utilized to support the rollout of AYA-focused models, such as youth peer navigators or dedicated site-level champions, thereby improving outcomes through mentorship, provider training, and quality improvement processes [52, 53]. These approaches demonstrate that facilitators are not only critical for knowledge translation but also serve as trusted intermediaries between youth, providers, and health systems. Given the similar structural and developmental challenges in youth TB care, future efforts should prioritize the incorporation of facilitator roles to coordinate the implementation of YFHS models, track fidelity, and foster intersectoral alignment. Investments in facilitation capacity, tailored to TB programs, will be necessary to ensure sustainable, scalable, and contextually appropriate integration of youth-centered care. Chiang and colleagues highlighted strategies to achieve this, including the establishment of a dedicated task force or working group within national TB programs to support and guide the implementation of YFHS [31]. Such a group should consist of youth advisors, as well as experts in youth TB and in the delivery of YFHS.

### Limitations

Several limitations should be considered when interpreting the findings of this review. First, the evidence base was limited in scope and methodological rigor, with most included studies qualitative or descriptive in design and relatively few intervention or implementation studies evaluating youth-friendly TB service models. Second, substantial heterogeneity in how YFHS concepts were defined and operationalized across studies limited direct comparability and synthesis across settings. Third, many studies recruited participants already engaged in care, potentially underrepresenting AYAs who were disengaged from TB services or unable to access care, including highly marginalized youth populations.

Although the search strategy was developed with a medical informationist and conducted across multiple databases, relevant studies indexed outside the selected databases or unpublished programmatic evaluations may not have been captured. In addition, despite inclusion of studies from multiple high- and medium-burden settings, the limited number of studies per country or region constrained our ability to assess contextual or regional differences systematically. Finally, despite the application of the i-PARIHS framework to structure interpretation of findings, the reviewed studies rarely explicitly incorporated implementation science frameworks or implementation outcomes, limiting the ability to draw conclusions regarding optimal implementation strategies for YFHS integration into TB care.

## Conclusion

Findings from this integrative review underscore the urgent need to adapt TB services to better meet the needs of AYAs. Despite growing recognition of AYAs as a key population in the TB response, current service models frequently overlook youth-specific preferences, structural vulnerabilities, and developmental needs. Applying the i-PARIHS framework, we identified implementation challenges and opportunities across the domains of innovation, recipients, context, and facilitation. Moving forward, embedding youth-friendly principles into TB care will require concerted investments in tailored service models, trained facilitators, adolescent-responsive policies, and meaningful youth engagement to ensure that TB programs are not only accessible but also acceptable, equitable, appropriate, and effective for young people.

## Electronic supplementary material

Below is the link to the electronic supplementary material.

Supplementary Material 1

Supplementary Material 2

## Data Availability

The data that support the findings of this study are available in the supplementary material of this article.

## Abbreviations

ART: Antiretroviral treatment
AYA: Adolescents and young adults
DOT: Directly observed therapy
HCW: Healthcare workers
HIV: Human immunodeficiency virus
i-PARIHS: Integrated Promoting Action on Research Implementation in Health Services
JBI: Joanna Briggs Institute
mHealth: Mobile health
SRH: Sexual and reproductive health
TB: Tuberculosis
TPT: Tuberculosis preventive treatment
WHO: World Health Organization
YFHS: Youth-friendly health services
