# Supplementary material for: Youth-friendly health services for tuberculosis care: an integrative review and implementation science synthesis
Source: BMC Health Serv Res. 2026 Jun 10;26:1136. doi: 10.1186/s12913-026-14844-0 (PMC13479411; doi:10.1186/s12913-026-14844-0)
Supplement: Supplementary file 1 [file 12913_2026_14844_MOESM1_ESM.pdf]

Search Terms

PUBMED
("Tuberculosis"[MeSH Terms] OR "tuberculo*"[Title/Abstract]) AND ("Adolescent Health Services"[MeSH Terms] OR "adolescent friendly"[Title/Abstract] OR "youth friendly"[Title/Abstract])

EMBASE
('tuberculosis'/exp OR 'tuberculo*':ab,ti,kw) AND ('child health care'/exp OR 'adolescent-friendly':ti,ab,kw OR 'adolescent friendly':ti,ab,kw OR 'youth-friendly':ti,ab,kw OR 'youth friendly':ti,ab,kw)

CINAHL
[(MH "Tuberculosis+") ORTI tuberculo* OR ABtuberculo*] AND [(MH "Adolescence+") ORTI adolesecen* OR ABadolesecen* OR (MH "Young Adult") OR TI"young adult*" OR AB"young adult*" OR TI youth* OR AB youth*]

WEB OF SCIENCE
[tuberculosis (All Fields) or TB (All Fields)] AND [adolescen* (All Fields) OR Youth (All Fields) OR adolescent friendly (All Fields) OR youth friendly (All Fields)]
